# Neural stem cells transplantation combined with ethyl stearate improve PD rats motor behavior by promoting NSCs migration and differentiation

**DOI:** 10.1111/cns.14119

**Published:** 2023-03-16

**Authors:** Jiapei Huang, Lan Yi, Xiaoxiao Yang, Qi Zheng, Jun Zhong, Sen Ye, Xican Li, Hui Li, Dongfeng Chen, Caixia Li

**Affiliations:** ^1^ School of Basic Medical Sciences Guangzhou University of Chinese Medicine Guangzhou Guangdong China; ^2^ Research Center of Integrative Medicine (Key Laboratory of Chinese Medicine Pathogenesis and Therapy Research), School of Basic Medical Sciences Guangzhou University of Chinese Medicine Guangzhou Guangdong China; ^3^ School of Information Science and Technology Guangdong University of Foreign Studies Guangzhou Guangdong China; ^4^ School of Pharmaceutical Sciences Guangzhou University of Chinese Medicine Guangzhou Guangdong China

**Keywords:** C‐C motif chemokine ligand 5, C‐C motif chemokine receptor 5, ethyl stearate, neural stem cells, Parkinson's disease

## Abstract

**Background:**

In recent years, the ability of neural stem cells (NSCs) transplantation to treat Parkinson's disease (PD) has attracted attention. However, it is still a challenge to promote the migration of NSCs to the lesion site and their directional differentiation into dopaminergic neurons in PD. C‐C motif chemokine ligand 5 (CCL5) and C‐C motif chemokine receptor 5 (CCR5) are expressed in the brain and are important regulators of cell migration. It has been reported that ethyl stearate (PubChem CID: 8122) has a protective effect in 6‐OHDA‐induced PD rats.

**Methods:**

Parkinson's disease rats were injected with 6‐hydroxydopamine (6‐OHDA) into the right substantia nigra, and striatum followed by 8 μL of an NSC cell suspension containing 100 μM ethyl stearate and 8 × 10^5^ cells in the right striatum. The effect of transplantation NSCs combined with ethyl stearate was assessed by evaluating apomorphine (APO)‐induced turning behavior and performance in the pole test. Quantitative real‐time reverse transcription–polymerase chain reaction (qRT‐PCR), Western blotting (WB), and immunofluorescence staining were also performed.

**Results:**

NSCs transplantation combined with ethyl stearate ameliorated the behavioral deficits of PD rats. PD rats that received transplantation NSCs combined with ethyl stearate exhibited increased expression of tyrosine hydroxylase (TH) and an increased number of green fluorescent protein (GFP)‐positive cells. Furthermore, GFP‐positive cells migrated into the substantia nigra and differentiated into dopaminergic neurons. The expression of CCL5 and CCR5 was significantly increased after transplantation NSCs combined with ethyl stearate.

**Conclusions:**

These findings suggest that NSCs transplantation combined with ethyl stearate can improve the motor behavioral performance of PD rats by promoting NSCs migration from the striatum to the substantia nigra via CCL5/CCR5 and promoting the differentiation of NSCs into dopaminergic neurons.

## Graphical abstract

Ethyl stearate enhances the expression of CCR5 in neural stem cells (NSCs), which contributes to promote the migration of grafted NSCs to the lesion site in PD model rats. Moreover, ethyl stearate induces NSCs directed differentiation into dopaminergic neurons, thus improving the movement disorder in PD model rats.

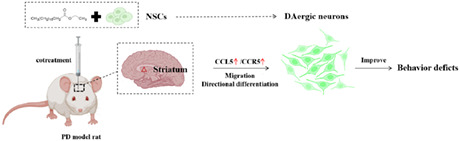

## 1 INTRODUCTION

Parkinson's disease (PD), as a common progressive neurodegenerative disease,^1^ is characterized by the progressive loss of nigrostriatal dopaminergic neurons.^2^ The central nervous system lacks repair mechanisms to replace lost dopaminergic neurons. Available treatments, which include pharmacological approaches, physiotherapy, deep brain stimulation, and stem cell‐based therapeutic approaches, can improve motor and nonmotor signs and symptoms.^3^ In recent years, there have been studies on the use of stem cell‐based therapeutic strategies to replace lost dopaminergic neurons in PD.^4^, ^5^, ^6^, ^7^ Neural stem cells (NSCs) exhibit the abilities of self‐renewal and differentiation potential, low immunogenicity, and good migratory ability in the context of transplantation,^8^, ^9^, ^10^, ^11^, ^12^, ^13^, ^14^, ^15^ thus, NSCs transplantation is one of the most promising methods for the treatment of PD.

However, most transplanted NSCs differentiate into glial cells rather than neurons. Additionally, in many studies, transplanted NSCs exhibit considerably limited migration ability, which restricts their therapeutic impact.^16^, ^17^, ^18^, ^19^ Chemokines are small secreted proteins with multiple physiological functions, and they have been shown to play a role in guiding the migration of neural progenitors in the developing brain^20^, ^21^. C‐C motif chemokine receptor 5 (CCR5), a seven‐transmembrane‐domain G‐protein‐coupled receptor expressed in the central nervous system, is stimulated by chemokine ligands CCL3, CCL4, and CCL5^.^ ^22^ Recently, studies have shown that NSCs can express CCR5.^23^, ^24^, ^25^, ^26^ Thus, NSCs can exhibit chemotactic responses to the relative chemokine ligands; however, few studies have reported the effect of CCL5/CCR5 on the migration of transplanted NSCs in PD.

In our previous studies, we found that plastrum testudinis extract (PTE) could improve the behavior of PD model rats and that its active ingredient was ethyl stearate. In this study, we treated NSCs with ethyl stearate and transplanted them into the striatum of PD model rats. The results showed that NSCs transplantation combined with ethyl stearate rescued the behavioral deficits in PD rats by promoting NSCs migration and differentiation.

## 2 METHODS AND MATERIALS

### 2.1 Animals

Specific‐pathogen‐free (SPF) male Sprague–Dawley (SD) rats weighing 180–220 g were used for in vivo experiments, and pregnant rats with 14‐ to 17‐day embryos were used for NSCs isolation. All experimental animals were provided by the Laboratory Animal Research Center of Guangzhou University of Chinese Medicine (ARCG‐UCM). The rats were housed at ARCG‐UCM at a temperature of 24–26°C and humidity of 40–60% on a 12/12‐hour light/dark cycle and provided access to food and water. The rats were allowed to acclimate to the laboratory environment for 1 week before the experiment was started. After the completion of the in vivo experiment, rats from each group were sacrificed for tissue collection via injection of sodium pentobarbital. Brains were removed rapidly from decapitated rats and cooled immediately in ice‐cold 0.9% NaCl solution. We removed the cortex and dissected the striatum and substantia nigra according to the rat brain atlas.

### 2.2 NSCs culture and preparation of green fluorescent protein (GFP)‐NSCs

Embryos were collected at embryonic days 14–17 from pregnant SD rats, and NSCs were isolated from the brains of the embryos by mechanical dissociation and trypsin digestion under aseptic conditions. The digestion of the dissected tissues was terminated by using DMEM/F12 (Gibco) containing 10% fetal bovine serum. After being washed twice with phosphate‐buffered saline (PBS, Gibco), the cells were plated in culture bottles in serum‐free DMEM/F12 supplemented with 20 ng/mL epidermal growth factor (EGF, GenScrip), 20 ng/mL basic fibroblast growth factor (bFGF, GenScrip) and 2% B27 (v/v, Gibco) as well as 100 units/mL penicillin and 100 μg/mL streptomycin (Gibco); they were then cultured at 37°C in an incubator containing 5% CO_2_. NSCs were cultured and passaged with serum‐free medium, and they were used at passage two in the subsequent experiment. Immunofluorescence staining, Western blotting (WB), quantitative real‐time reverse‐PCR (qRT‐PCR), and analysis of NSC migration were performed.

For the preparation of GFP‐NSCs, the single NSC was transfected with eGFP‐expressing lentiviral vectors (MOI = 100; LVCON077, Genechem) for 7 days with HitransG A (REVG003, Genechem).

### 2.3 In vitro experiment

NSCs in passage three neurospheres cultured in proliferation medium were identified by immunofluorescence staining for the marker Nestin. The neurospheres were divided into different groups and uniformly seeded in 6‐well culture plates. Then they were stimulated with different doses of ethyl stearate. The groups were the blank control group (0 μM group), 10 μM group, 100 μM group, and 1000 μM group. The cell migration distance and expression of CCR5 in these groups were evaluated.

For in vitro differentiation, NSCs were dissociated into single‐cell suspensions in DMEM/F12 supplemented with 10% fetal bovine serum as well as 100 units/mL penicillin and 100 μg/mL streptomycin and then plated in 6‐well culture plates. Different doses of ethyl stearate were added to induce cell differentiation. After 5 days of induction, Immunofluorescence staining, WB analysis, and qRT‐PCR were performed.

### 2.4 Quantification of the cell migration distance

Neurospheres were uniformly plated in poly‐lysine‐coated 6‐well plates at a low density (10–15 neurospheres per dish) to ensure a large distance between the individual spheres and cultured in the proliferation medium. The neurospheres were then treated with ethyl stearate (0, 10, 100, or 1000 μM) for 1–3 days, and the migratory behavior of NSCs was evaluated by measuring the end‐to‐end distance of the extended processes. The lengths of the 10 longest processes were estimated from the edge of the neurosphere or cell body to the tip of the processes. Distance measurements were performed by using ImageJ.^27^

### 2.5 PD model preparation and analysis of apomorphine (APO)‐induced rotation

Specific‐pathogen‐free male SD rats were randomly divided into two groups: the sham‐operated group and the model group. Rats in the model group received unilateral stereotaxic injections of 8 μL 6‐hydroxydopamine (6‐OHDA, 4 μg/μl, H873296, Macklin, Shanghai, China) in the substantia nigra and striatum as previously described.^28^ The following coordinates were used to target the right substantia nigra and striatum: AP = −5.7 mm, L = ‐2.0 mm, DV = ‐8.7 mm; AP = +0.5 mm, L = ‐2.5 mm, DV = ‐7.0 mm. Rats were anesthetized with isoflurane and then placed in a stereotaxic frame (RWD). Animals in the sham‐operated group were injected with the same volume of 0.9% NaCl solution at the same coordinates. Beginning a week after surgery, the rats were intraperitoneally injected with APO (0.5 mg/kg, 017–18,321, Wako, Japan) once a week, and rotation behavior was assessed for 30 min. The model establishment was considered successful if the rats that were injected with 6‐OHDA exhibited more than 6 rotations per minute within 6 weeks of observation.

### 2.6 Transplantation and behavioral test

Model rats were randomly distributed into three groups: the model group, the NSCs group, and the ethyl stearate+NSCs group (abbreviated as “ethyl stearate group”). Before transplantation, NSCs were transfected with lentivirus expressing GFP for 7 days and then dissociated into a single‐cell suspension. The rats were anesthetized with isoflurane and immobilized in a stereotaxic frame. Animals in the ethyl stearate group received an injection of 8 μL of cell suspension (approximately 8 × 10^5^ cells in 8 μL of phosphate‐buffered saline (PBS) containing 100 μM ethyl stearate) in the ipsilateral striatum (AP = +0.5 mm, L = ‐2.5 mm, DV = ‐7.0 mm). Animals in the NSCs group were injected with the same number of cells in the same volume at the same site. Animals in the model group were injected with the same volume of PBS into the same site. Beginning 1 week after transplantation, behavioral testing was carried out for 6 weeks. The APO‐induced rotation test was performed as described above.

Before rotation induction, the rats were placed in a head‐down position at the top of a 60 cm long, 1 cm diameter vertical wooden pole placed in a cage. The wooden poles were wrapped with medical gauze to ensure sufficient friction. The time required for the rats to climb from the top of the pole to the bottom was recorded. Each rat performed the test 3 times at an interval of 5 s, and the average value was taken. Before the formal experiment, the rats underwent two rounds of training to adapt to the protocol.^29^

### 2.7 qRT‐PCR

Total RNA was extracted from 2 × 10^6^ cells or 25 mg of rat brain tissue with TRIzol reagent (No. 15596026 Invitrogen). According to the manufacturer's instructions, the RNA was reverse transcribed into cDNA with the PrimeScript™ RT Reagent Kit (No. RR036A, Takara). qRT‐PCR was carried out with the TB Green™ Premix Dimer Eraser™ kit (No. RR820B, Takara) according to the following protocol: initial denaturation at 95°C for 30 s, followed by 40 cycles of 95°C for 5 s, 58°C for 30 s, and 72°C for 1 min. Relative gene expression was analyzed by the 2^−ΔΔCq^ method. Primers for GAPDH, which was used as a housekeeping gene for the evaluation of mRNA expression levels, were purchased from Sangon Biotech (B661204‐0001). The primer sequences are shown in Table 1.

**TABLE 1:** Primers for qRT–PCR.

Gene	Sequence
Th‐Forward	ACTGTGGCTACCGAGAGGAC
Th‐Reverse	AATCACGGGCGGACAGTAGA
Ccl5‐Forward	GACACCACTCCCTGCTGCTTTG
Ccl5‐Reverse	CTCTGGGTTGGCACACACTTGG

### 2.8 Immunofluorescence staining

Six weeks after transplantation, the rats were sacrificed by injection of sodium pentobarbital, and the brain tissues were fixed with 4% paraformaldehyde. After being immersed in 15% and 30% sucrose in PBS, the brain tissues were sliced with a freezing microtome (20 μm thick). Cultured cells were fixed with 4% paraformaldehyde in PBS for 20 min at room temperature. The fixed cells and brain sections were then permeabilized with 0.5% Triton X‐100 for 15 min at 37°C, blocked in 10% goat serum for 30 min at 37°C, and incubated overnight at 4°C with primary antibodies. They were then incubated with secondary antibodies in the dark at room temperature for 1 h. Then, the stained samples were counterstained with DAPI (P0131, Beyotime Biotechnology) and observed with a laser scanning confocal microscope. Images were taken with ZEN 2 software. The following antibodies were used: Nestin (1:50, abs137231, Absin, China), NSE (1:50, BA0535, Boster), GFAP (1:50, BA0056, Boster), CCR5 (1:50, sc‐17,833, Santa Cruz Biotechnology), TH (1:500, ab112, Abcam), CCL5 (1:50, sc‐365,826, Santa Cruz Biotechnology), goat anti‐rabbit IgG H&L (Alexa Fluor® 488,1:1000, ab150077, Abcam), goat anti‐mouse IgG H&L (Alexa Fluor® 647,1:1000, ab150119, Abcam), and goat anti‐rabbit IgG H&L (Alexa Fluor® 647,1:1000, ab150079, Abcam).

### 2.9 WB analysis

Total protein extracts were separated by 10% SDS–PAGE (Beyotime) and transferred onto polyvinylidene difluoride (PVDF) membranes, which were blocked with 5% nonfat milk for 2 h and then incubated with antibodies against alpha‐synuclein (1:500, No. ab212184, Abcam), TH (1:500, No. ab112, Abcam), Gapdh (1:1000, 14C10, CST), CCR5 (1:200, sc‐17,833, Santa Cruz Biotechnology), and CCL5 (1:100, sc‐365,826, Santa Cruz Biotechnology) antibodies at 4°C overnight. Then, the membranes were washed with TBST and incubated with goat anti‐rabbit IgG H&L (HRP‐conjugated) (1:10000, ab6721, Abcam) or rabbit anti‐mouse IgG H&L (HRP‐conjugated) (1:10000, ab6728, Abcam) for 1 h at room temperature. The signal was detected with Immobilon Western Chemiluminescent HRP Substrate (Millipore), and the gray values of the bands were analyzed with ImageJ software.

### 2.10 Statistical analysis

GraphPad Prism 8.0 software was used for statistical analysis, and the data for each group are presented as the mean ± standard deviation of at least three independent experiments. The Shapiro–Wilk test was used to test the normal distribution, and Levene's test was used to test the homogeneity of variance. Then, the data were analyzed by Student's‐*t* test, Mann–Whitney *U* test, or one‐way analysis of variance followed by Tukey's test or the Dunnett T3 test. A *p*‐value <0.05 was considered significant.

## 3 RESULTS

### 3.1 Identification of NSCs and preparation of GFP‐NSCs

**FIGURE 1:**
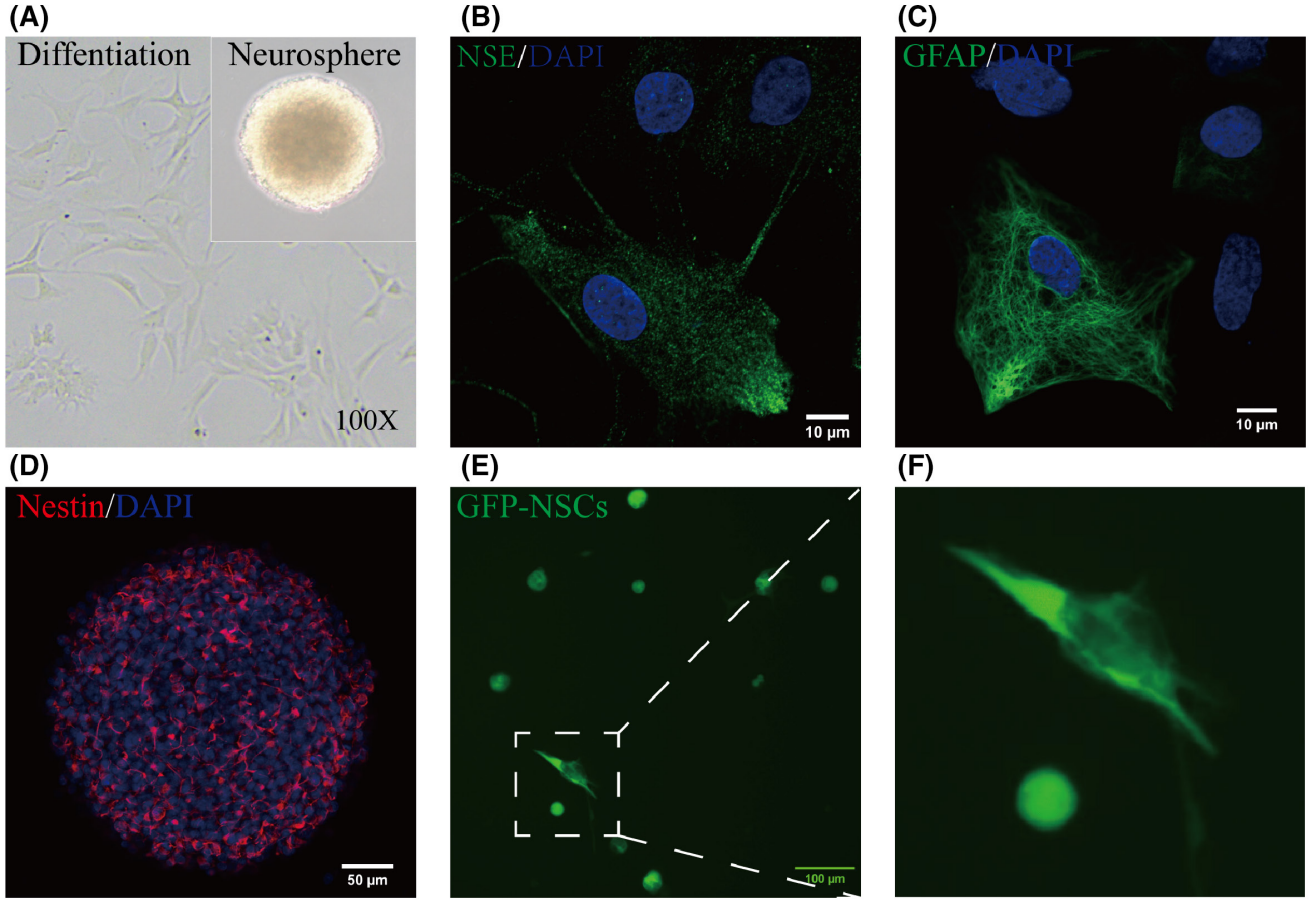
Identification of NSC and construction of GFP‐NSCs. (A) Neurospheres were cultured for 7 days in the proliferation medium. NSCs that adhered to the plate and differentiated in the differentiation medium were observed under a light microscope (100×). (B) After 5 days of culture in the differentiation medium, cells that were immunopositive for NSE (green) were observed. DAPI (blue) was used to stain nuclei (scale bar: 10 μm). (C) Cells immunopositive for GFAP (green) were observed. DAPI (blue) was used to stain nuclei (scale bar: 10 μm). (scale bar: 10 μm) (D) Neurospheres were positive for Nestin (red), and the nuclei were stained with DAPI (blue). (scale bar: 50 μm) (E, F) GFP was detected in neurospheres and differentiated cells after transfection with lentiviral vectors.

After being cultured for 5 days, the cells formed large spherical colonies, which were immunopositive for Nestin (Figure 1A,D). Later, these neurospheres were collected and dissociated into single cells. The single cells adhered to the plate and showed a long fusiform shape with prominent protuberances (Figure 1A). The cells derived from NSCs were stained with neuron‐specific enolase (NSE, a marker of neurons) and glial fibrillary acidic protein (GFAP, a marker of astroglia) (Figure 1B,C). Then, the NSCs were transfected with lentiviral vectors expressing GFP for subsequent experiments. After the transfected cells were cultured for 5 days, GFP was detected in the neurospheres and differentiated cells (Figure 1E,F). The above results illustrate that we successfully isolated and transfected NSCs.

### 3.2 Ethyl stearate promotes NSCs differentiation into dopaminergic neurons and NSCs migration in vitro

**FIGURE 2:**
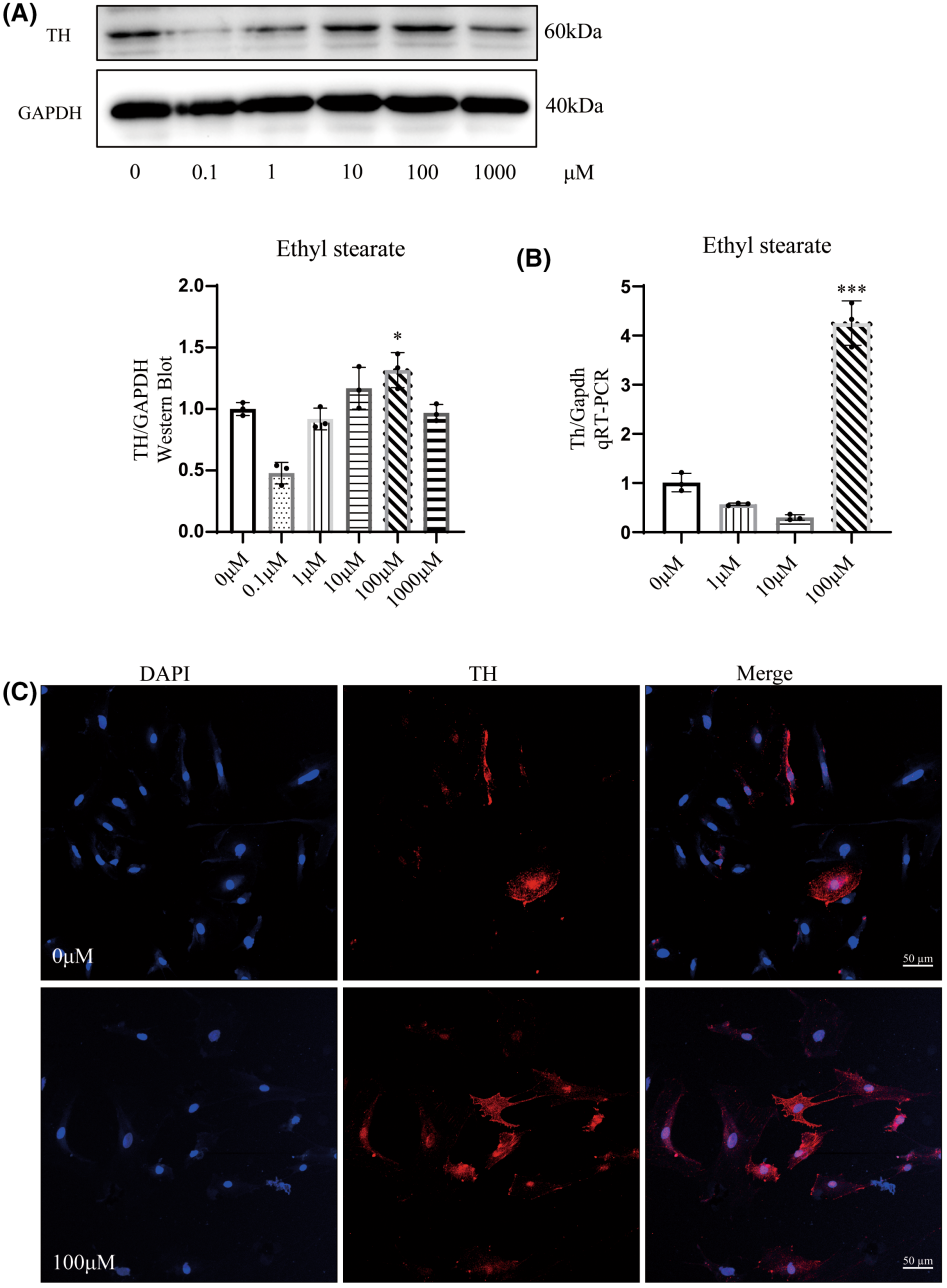
Effect of ethyl stearate on the differentiation of NSCs. (A) The expression of TH (60 kDa) was examined by WB. (B) qRT‐PCR analysis was used to measure the mRNA expression of Th. (C) Cells immunopositive for TH (green) were observed. DAPI (blue) was used for staining nuclei. (scale bar: 50 μm) Three independent experiments were conducted. The data are presented as the mean ± SEM. *p < 0.05, and ***p < 0.001 compared with the 0 μM group, one‐way analysis of variance followed by Tukey test. (*n* = 3)

To confirm that ethyl stearate could promote NSCs differentiation into dopaminergic neurons, neurospheres were dissociated into single cells for the following experiment. Five days after ethyl stearate‐induced NSCs differentiation, we assessed TH expression, which is considered as a biomarker related to dopaminergic neurons.^2^ WB showed that TH protein expression was significantly increased in the 100 μM group (Figure 2A). Subsequently, we used qRT‐PCR to measure the Th mRNA level in the different groups. The results showed that Th mRNA expression was significantly upregulated in the 100 μM group compared with the 0 μM group (Figure 2B). Moreover, there were more TH‐positive cells in the 100 μM group (Figure 2C).

**FIGURE 3:**
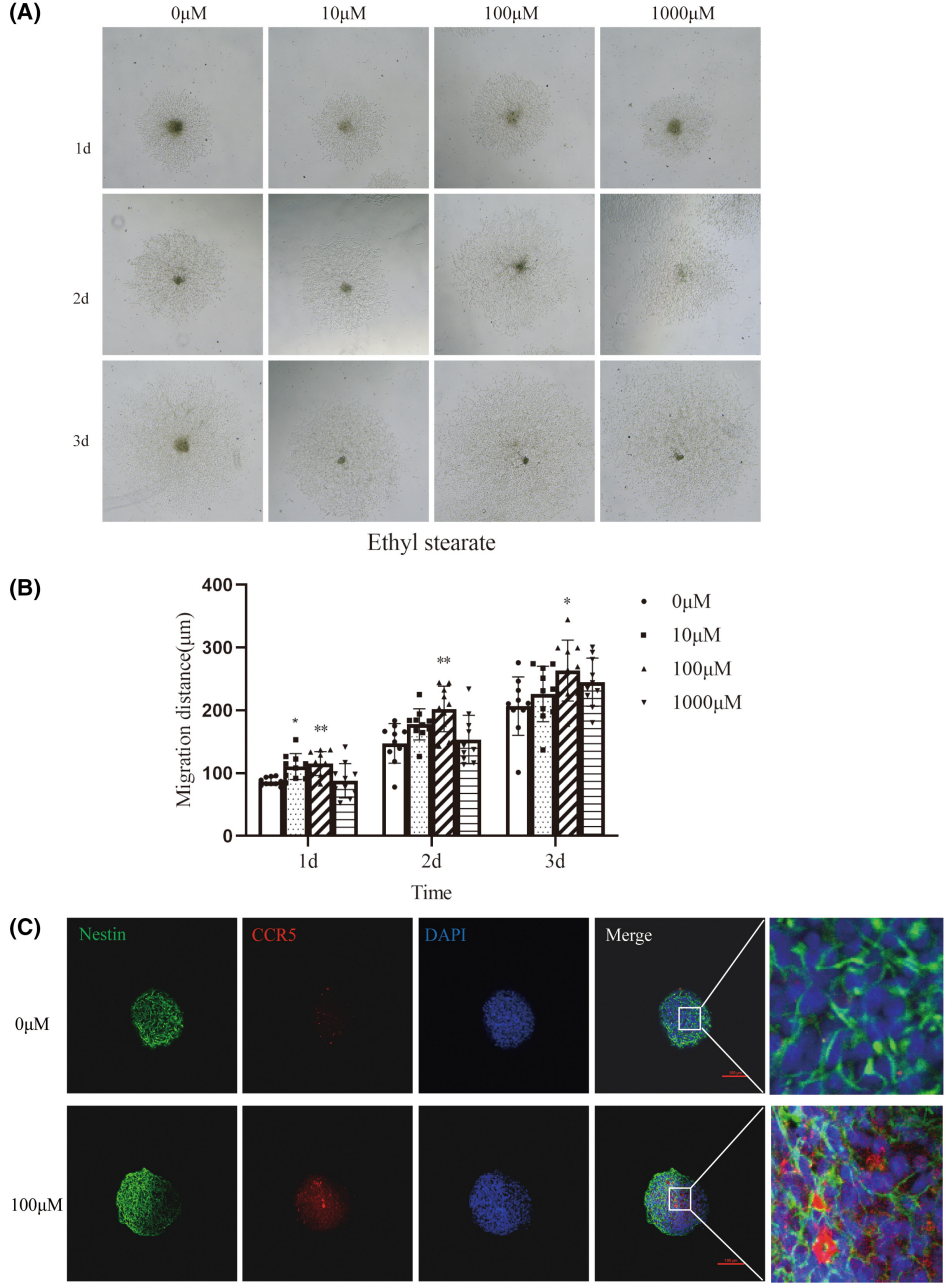
Effect of ethyl stearate on the migration of NSCs. (A, B) Cell migration was observed under a light microscope (100×). The distance traveled by the cells was assessed 1–3 days after the neurospheres were plated (*n* = 10). (C) CCR5 expression in Nestin‐positive cells was assessed. Staining for nestin (green), CCR5 (red), and DAPI (blue) is shown (scale bar: 100 μm). Nestin (green) was used to label the NSCs. DAPI (blue) was used to label the nuclei. Three independent experiments were conducted. The data are presented as the mean ± SEM. *p < 0.05 and **p < 0.01 compared with the 0 μM group. Day 1: one‐way analysis of variance followed by the Dunnett‐T3 test. Day 2 and Day 3:one‐way analysis of variance followed by Tukey test.

Next, to examine whether ethyl stearate can promote NSCs migration, the distance traveled by the cells was measured. The cells were observed to migrate out from the neurospheres, and 100 μM ethyl stearate treatment significantly increased the migration distance (Figure 3A,B). Moreover, the immunofluorescence staining results showed that there were more CCR5‐positive cells in the 100 μM ethyl stearate group (Figure 3C).

These results suggested that 100 μM ethyl stearate promoted NSCs differentiation into dopaminergic neurons and promoted NSCs migration to lesions by enhancing the expression of CCR5, indicating that NSCs stimulated with 100 μM ethyl stearate might have therapeutic potential as a cell replacement strategy in PD rats.

### 3.3 PD model rats exhibit motor deficits and loss of dopaminergic neurons

**FIGURE 4:**
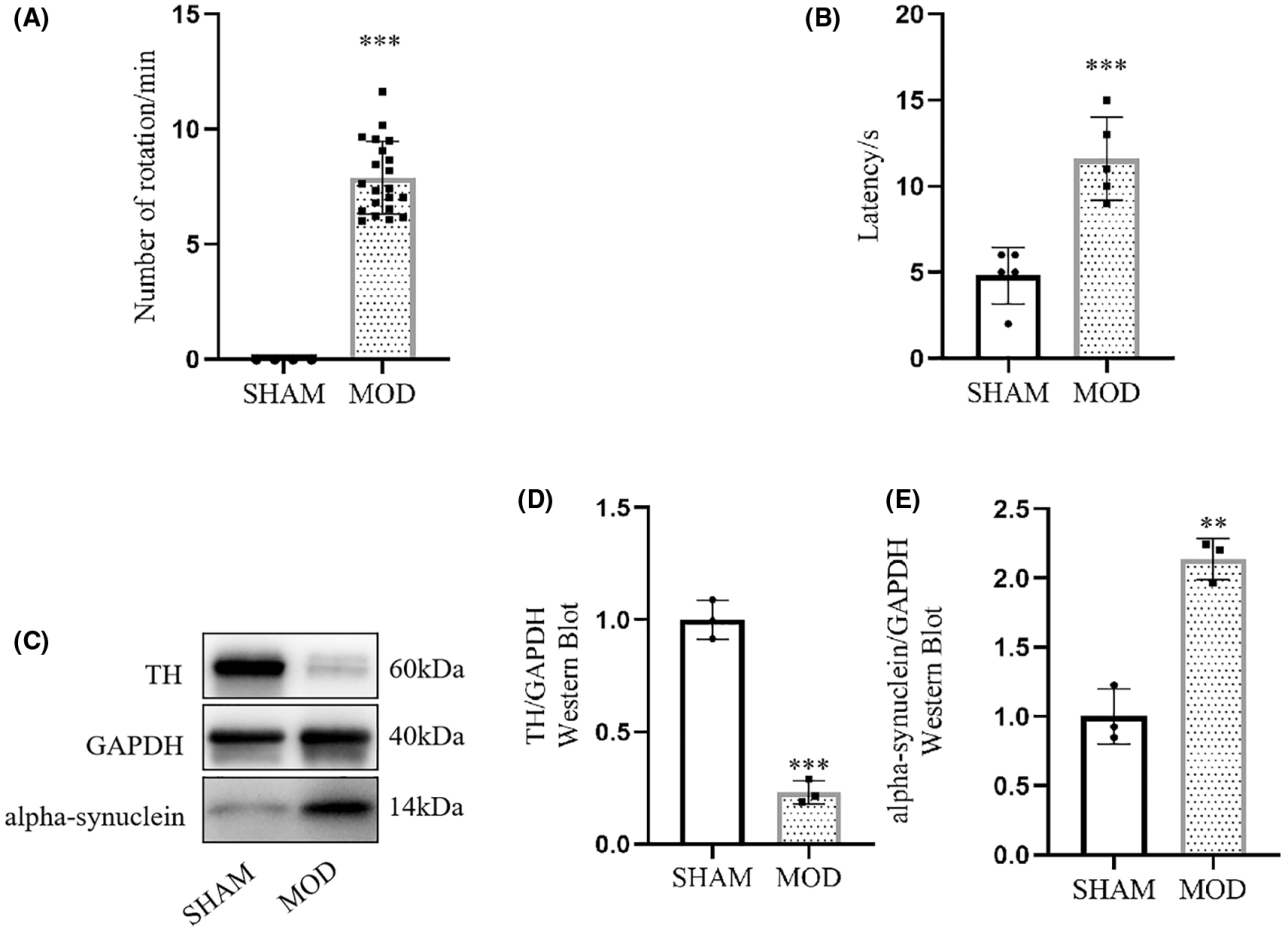
Behavior of PD rats and the protein expression of TH and alpha‐synuclein in PD rats in substantia nigra. (A) APO‐induced rotation behavior of the sham‐operated group (*n* = 5) and the model group (*n* = 21). (B) Performance in the pole test. The time required to climb down the pole was recorded for the sham‐operated group and the model group. (*n* = 5) (C–E) TH and alpha‐synuclein protein levels in substantia nigra in the sham‐operated group and the model group. (*n* = 3) (A) ***p < 0.001 compared with the sham group, Mann–Whitney *U* test. (B, D, and E): **p < 0.01, *** *p* < 0.001 compared with the sham group, unpaired Student's *t*‐test.

Based on the in vitro results, we next examined whether transplantation NSCs combined with 100 μM ethyl stearate could alleviate behavioral deficits in PD rats. First, we constructed a rodent model of PD by stereotactic injection of 6‐OHDA and identified PD model rats through analysis of APO‐induced rotations. The animals in the sham group did not exhibit rotation behavior. However, the rats in the model group showed rotation behavior, completing more than 6 rotations per minute (Figure 4A).

Moreover, the pole‐climbing experiment was conducted to assess the limb coordination of the rats. The results showed that the rats in the sham group climbed down the pole more quickly and smoothly than the PD model rats. The PD rats took a significantly longer time than the rats in the sham group to climb down the pole and showed a lack of motor coordination during pole climbing (Figure 4B). Then, WB confirmed that TH expression was significantly decreased and alpha‐synuclein expression was obviously increased in the PD model group compared with the sham‐operated group (Figure 4C–E). The above results indicated that the PD rat model was successfully constructed.

### 3.4 NSCs Transplantation combined with ethyl stearate rescues behavioral deficits in PD rats

**FIGURE 5:**
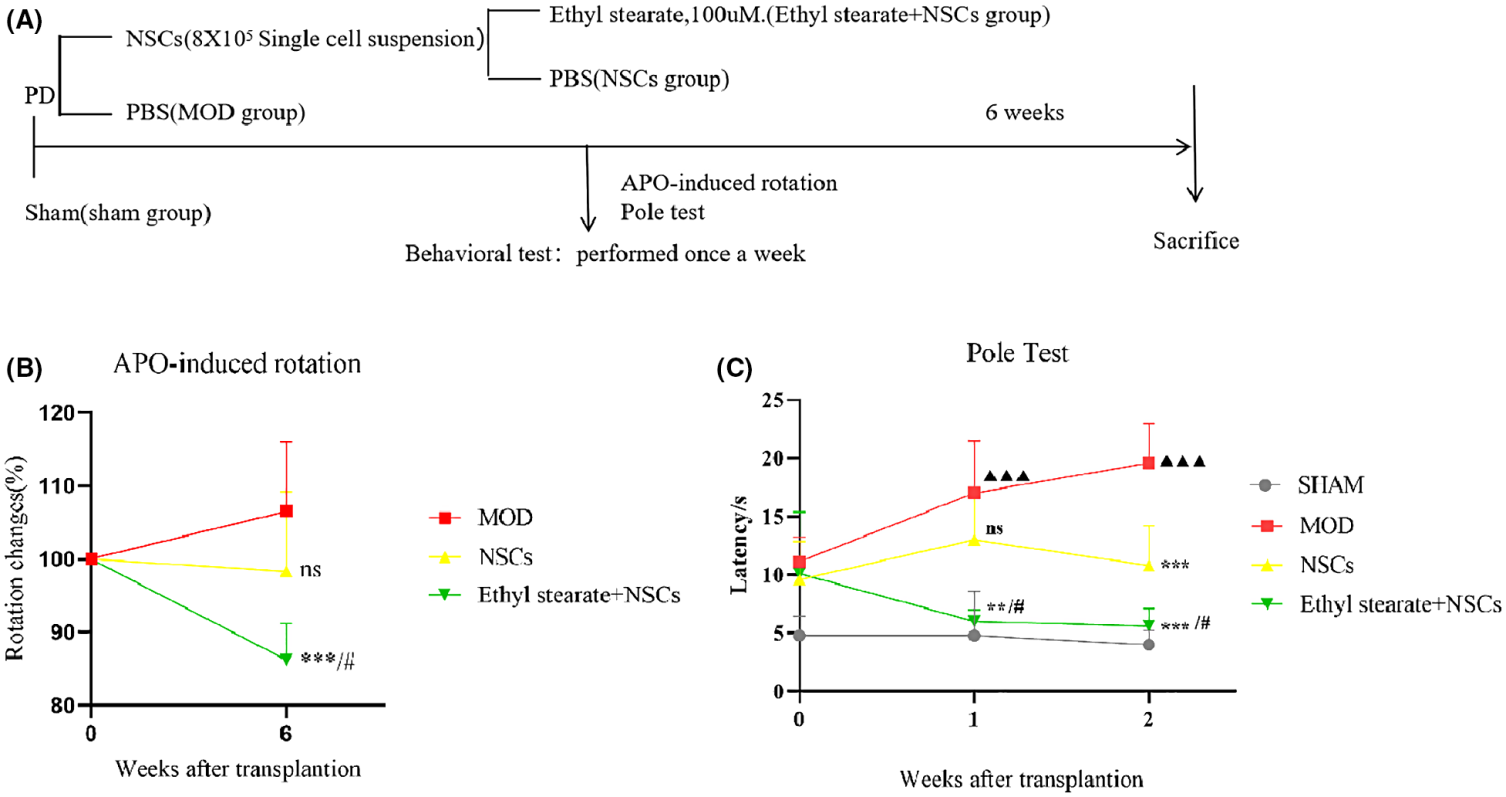
Behavior was analyzed after transplantation NSCs combined with ethyl stearate. (A) Diagram of the in vivo experiments. (B) Changes in APO‐induced rotation behavior in the model group, NSCs group, and ethyl stearate+NSCs group were analyzed after transplantation. (*n* = 4) (C) The pole test was used to test the motor coordination of the sham‐operated group, model group, NSCs group, and ethyl stearate+NSCs group after transplantation. (*n* = 5) The data are presented as the mean ± SEM. ns: *p* > 0.05, ** *p* < 0.01 and *** *p* < 0.001 compared with the model group; # *p* < 0.05, NSCs group versus ethyl stearate+NSCs group▲▲▲<0.001 model group versus sham group, one‐way analysis of variance followed by Tukey test.

Parkinson's disease model rats underwent stereotaxic injection of GFP‐labeled NSCs or GFP‐labeled NSCs and 100 μM ethyl stearate in the ipsilateral striatum. Animals in the model group were injected with the same volume of PBS at the same site. The protocol of the in vivo experiments is shown in Figure 5A.

Six weeks after surgery, APO‐induced rotation behavior was alleviated in the PD rats in the NSC group and especially the ethyl stearate group; however, this change was not observed in the animals injected with PBS. Importantly, rotation behavior was decreased to a greater extent in the ethyl stearate group than in the NSCs group (Figure 5B).

In addition, the pole test was used to assess the coordination of PD rats after transplantation. The results showed that 1 week after transplantation, the rats in the PD model group climbed the pole more slowly than the rats in the sham‐operated group. The average time to climb down the pole was significantly longer in the model group than in the sham group. The rats in the NSCs group and ethyl stearate group exhibited better performance in the pole climbing test. After 2 weeks of treatment, the rats in the NSCs group and ethyl stearate group were able to climb down the pole faster than the rats in the model group. The pole climbing time of the rats in the ethyl stearate group was similar to that of the rats in the sham group, suggesting that transplantation NSCs combined with ethyl stearate could significantly alleviate the motor behavior deficits of PD rats (Figure 5C).

### 3.5 NSCs Transplantation combined with ethyl stearate protects dopaminergic neurons in PD model rats

**FIGURE 6:**
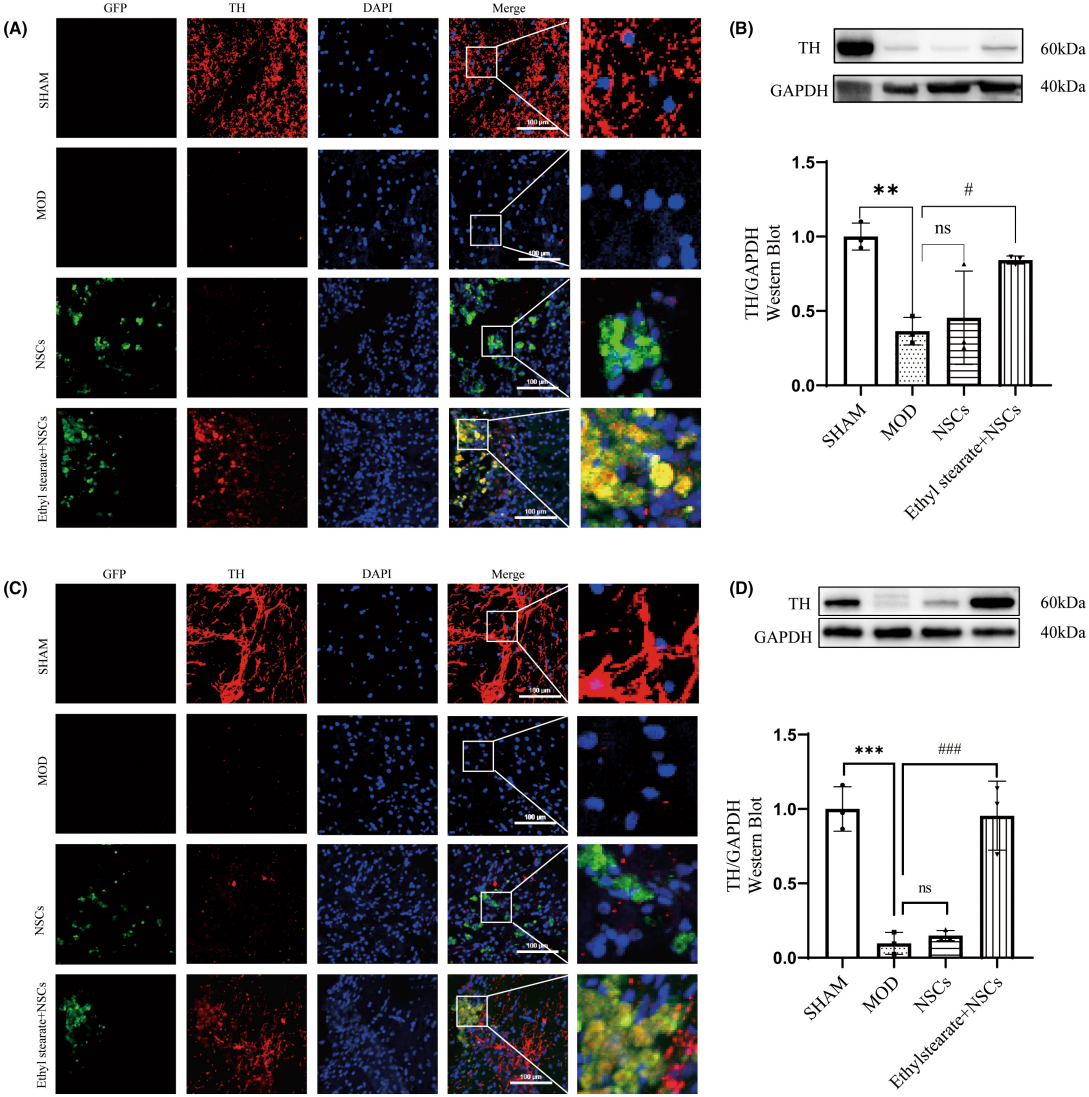
Expression of TH after transplantation NSCs combined with ethyl stearate in the striatum and the substantia nigra. (A) Immunofluorescence staining showed TH (red) and GFP (green) positive cells in the striatum. DAPI (blue) was used to stain nuclei. (B) Protein levels of TH in the striatum. (*n* = 3 per group) (C) Immunofluorescence staining showed TH (red) and GFP(green) positive cells in the substantia nigra. DAPI (blue) was used to stain nuclei. (D) Protein levels of TH in the substantia nigra. (*n* = 3) The data are presented as the mean ± SEM. **p < 0.01 and *** *p* < 0.001 versus the sham group; ns: *p* > 0.05;#*p* < 0.05 and ###*p* < 0.001 versus the model group, one‐way analysis of variance followed by Tukey test. Scale bar: 100 μm.

To assess whether the improvement in motor function observed in the ethyl stearate group was related to the protection of dopaminergic neurons, we performed immunofluorescence staining and WB to measure the expression of TH in both the striatum and the substantia nigra. Immunofluorescence staining in the striatum showed that the number of TH‐positive cells was decreased in the model group compared with the sham group. However, the number of TH‐positive cells was markedly increased in the ethyl stearate group compared with the model and NSCs groups, and more GFP‐positive cells differentiated into TH‐positive cells in the ethyl stearate group, which implied that ethyl stearate promoted the differentiation of transplanted NSCs into dopaminergic neurons (Figure 6A). In addition, WB analysis showed that the protein expression of TH was increased after transplantation NSCs combined with ethyl stearate (Figure 6B), which indicated that ethyl stearate alleviated damage to dopaminergic neurons in PD rats.

In the substantia nigra, consistent with the results described above, TH‐positive cells in the ethyl stearate group were significantly increased compared with those in the model group and NSCs group. Interestingly, GFP‐positive cells appeared in the substantia nigra and differentiated into TH‐positive cells (Figure 6C). WB also showed that the protein expression of TH was increased after the transplantation of NSCs combined with ethyl stearate (Figure 6D).

Taken together, these results implied that ethyl stearate promoted NSCs migration as well as the differentiation of NSCs into dopaminergic neurons in vivo.

### 3.6 Localization of GFP‐NSCs in PD model rats after NSCs transplantation combined with ethyl stearate in the ipsilateral striatum

**FIGURE 7:**
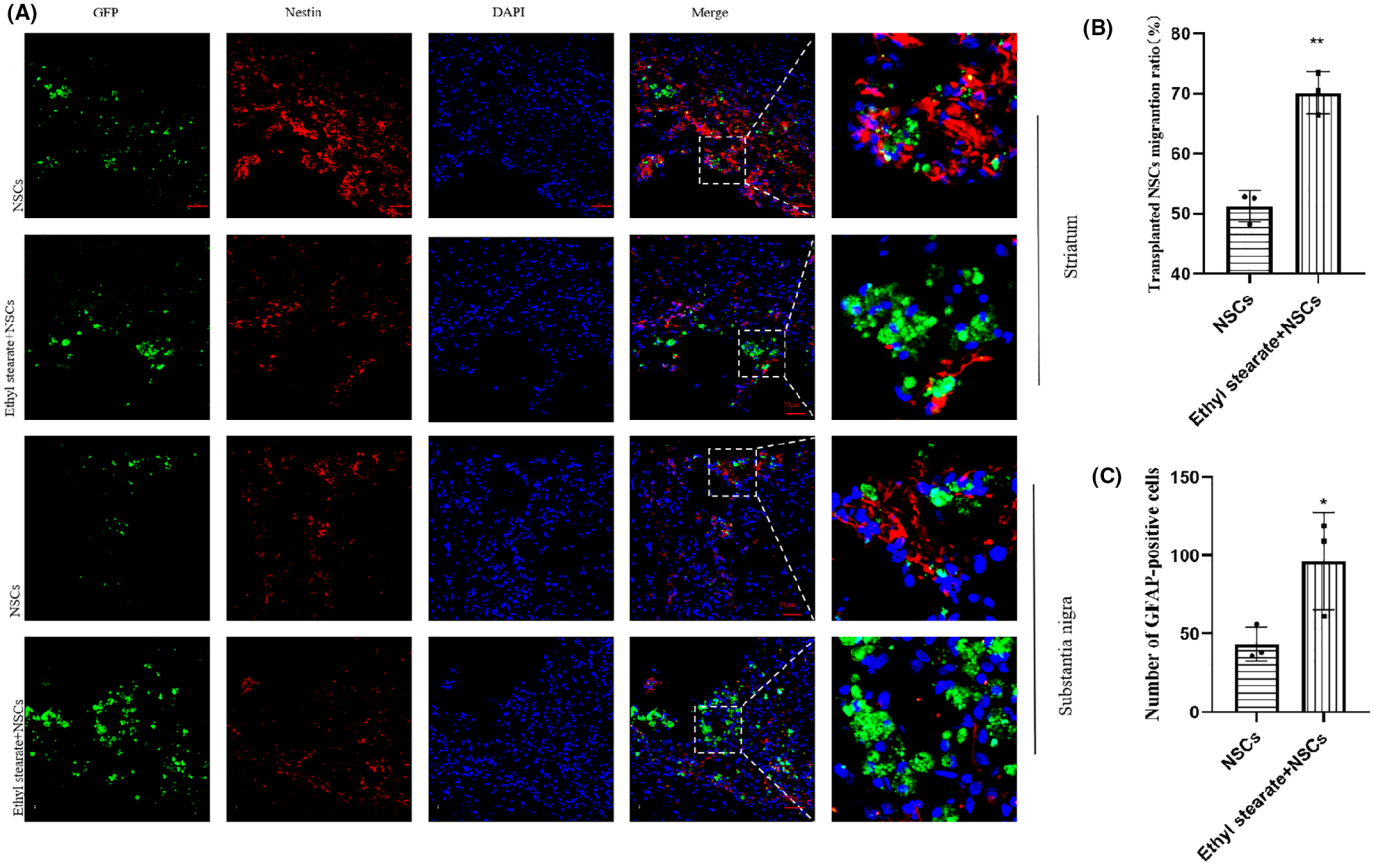
Immunofluorescence analysis of the position and survival of transplanted cells 6 weeks after transplantation. (A) Immunofluorescence staining showed Nestin (red) and GFP (green) positive cells in the striatum and the substantia nigra. DAPI (blue) was used to stain nuclei. (B) The analysis of the migration of grafted cells from the striatum to the substantia nigra. (C) The number of GFP‐positive cells in both the striatum and substantia nigra. The data are presented as the mean ± SEM (*n* = 3). * *p* < 0.05 and ** *p* < 0.01 versus the NSCs group, unpaired Student's *t*‐test. Scale bar: 50 μm.

Furthermore, we detected GFP‐positive cells in both the striatum and the substantia nigra at 6 weeks after transplantation by immunofluorescence staining. GFP‐positive cells were observed in both the striatum and the substantia nigra (Figure 7A), indicating that the transplanted NSCs migrated to the substantia nigra. Next, we calculated the proportion of GFP‐positive cells that migrated from the striatum to the substantia nigra and the total number of GFP‐positive cells. The number of GFP‐positive cells in the substantia nigra was increased in the ethyl stearate group compared with the NSCs group. The proportion of GFP‐positive cells that migrated from the striatum to the substantia nigra was increased in the ethyl stearate group (Figure 7B). In addition, the number of GFP‐positive cells was significantly higher in the ethyl stearate group than in the NSCs group (Figure 7C), indicating that ethyl stearate promoted the survival and migration of transplanted NSCs.

### 3.7 Ethyl stearate increases the expression of CCL5 and CCR5 in a PD rat model

**FIGURE 8:**
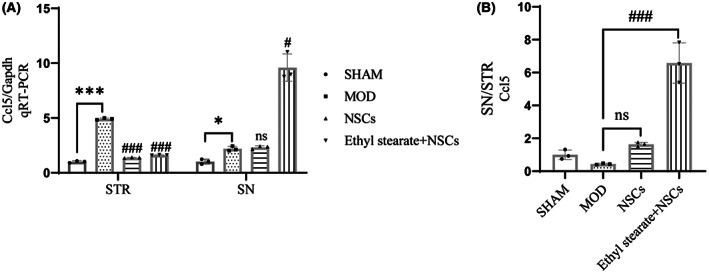
After transplantation NSCs combined with ethyl stearate, the mRNA levels of Ccl5 in the striatum and substantia nigra. (A) qRT‐PCR was performed to measure the mRNA levels of Ccl5 in the striatum and substantia nigra. (B) The expression differences of Ccl5 between the striatum and substantia nigra. The data are presented as the mean ± SEM (*n* = 3). *p < 0.05 and **p < 0.01 versus the sham group; ns: *p* > 0.05, # *p* < 0.05, and ### *p* < 0.001 versus the model group, one‐way analysis of variance followed by Tukey test.

We measured the expression of the related chemokine CCL5 ex vivo. qRT‐PCR results showed that Ccl5 expression was upregulated in the striatum and substantia nigra in the model groups compared with the sham group. The mRNA expression of Ccl5 decreased after NSCs transplantation combined with ethyl stearate in the striatum compared with the model group but increased in the substantia nigra (Figure 8A), which showed significant expression differences between the striatum and substantia nigra in the ethyl stearate group (Figure 8B).

**FIGURE 9:**
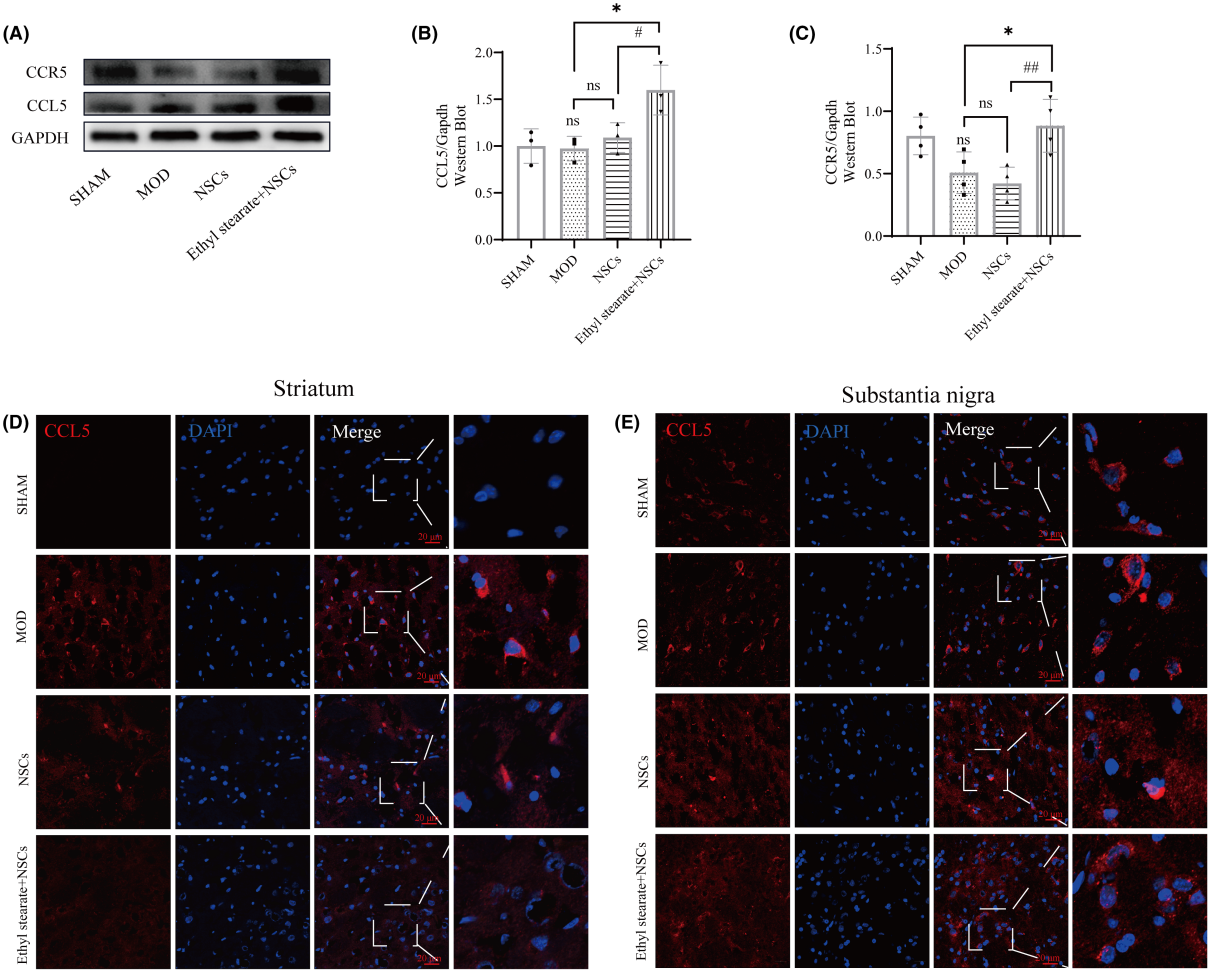
After transplantation NSCs combined with ethyl stearate, the protein levels of CCL5 and CCR5 in the striatum and the substantia nigra. (A–C) Western blot analysis of CCL5 and CCR5 protein levels in the substantia nigra. (*n* = 3–4) (D, E) Immunofluorescence staining of CCL5 in the striatum and the substantia nigra. The red fluorescence represents CCL5, and the blue fluorescence indicates the nuclei. The data are presented as the mean ± SEM. ns: *p* > 0.05 versus the sham group; ns: *p* > 0.05 and * *p* < 0.05 versus the model group; # *p* < 0.05, and ## *p* < 0.01 versus the NSC group, one‐way analysis of variance followed by Tukey test. Scale bar: 20 μm.

**FIGURE 10:**
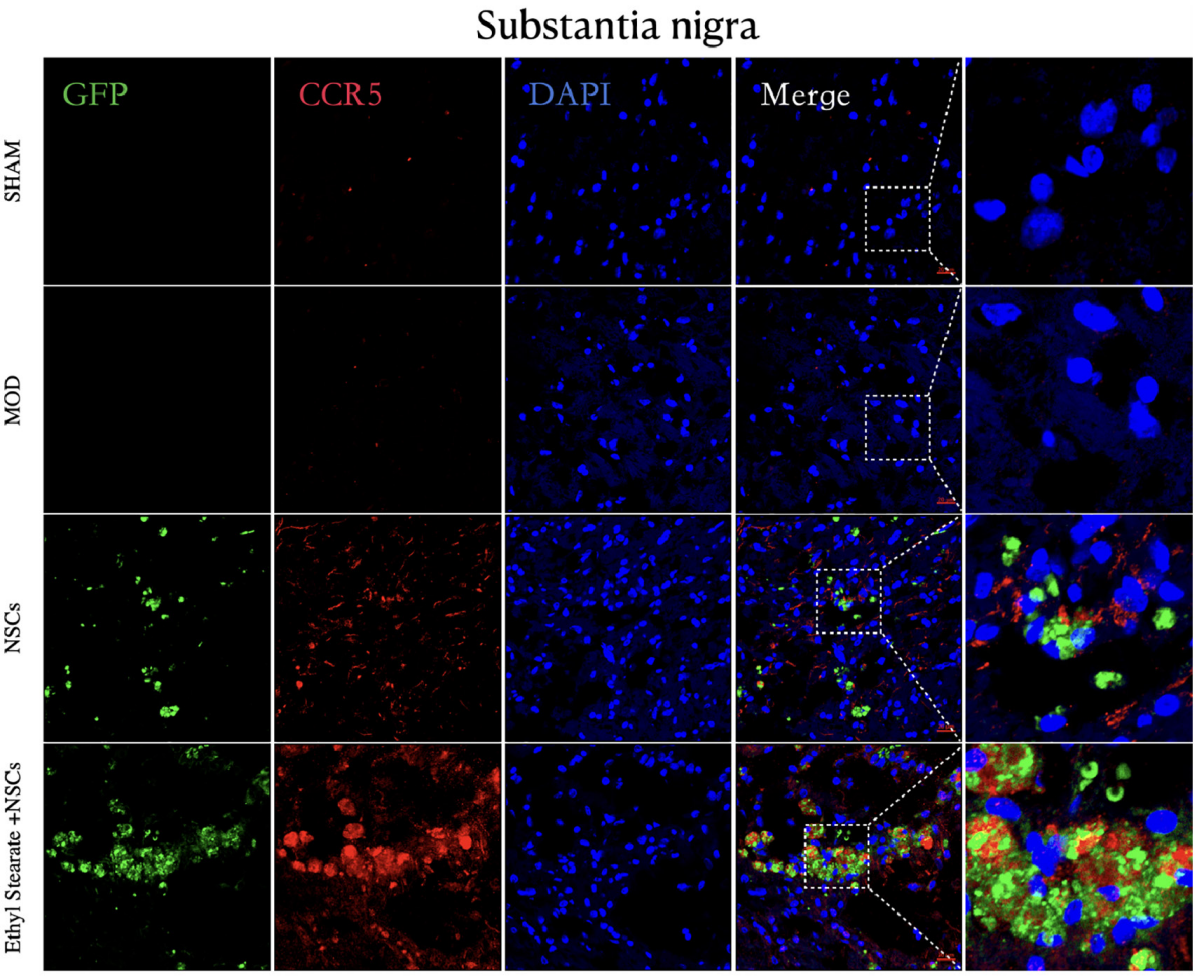
Immunofluorescence staining of CCR5 in the substantia nigra after transplantation. The green fluorescence represents transplanted cells, the red fluorescence represents CCR5, and the blue fluorescence indicates the nuclei. These experiments were conducted in three independent replicates. Scale bar: 20 μm.

The protein expression of CCL5 and CCR5 in the substantia nigra was upregulated in the ethyl stearate group compared with the model group and the NSCs group (Figure 9A). Immunofluorescence staining confirmed the difference in CCL5 expression between the striatum and substantia nigra in the ethyl stearate group (Figure 9D,E). Furthermore, the expression of CCR5 in NSCs labeled with GFP in the substantia nigra was significantly increased in the ethyl stearate group compared with the NSCs group (Figure 10), indicating that CCL5 induced CCR5‐NSCs to migrate to the lesion. Taken together, these findings suggest a potential mechanism by which NSCs transplantation combined with ethyl stearate can ameliorate PD.

## 4 DISCUSSION

PD is related to various factors, such as age, the environment, genetics, and abnormal alpha‐synuclein accumulation. At present, treatments for PD are still limited. Treatment for PD is mainly based on drug therapy. Levodopa (L‐DOPA), the mainstay drug for treating PD, can improve motor and nonmotor signs and symptoms, but it may lead to dyskinesia after a few years.^30^, ^31^ In light of the current situation, the NSC‐based replacement strategy is regarded as an extremely promising therapeutic approach for PD.^32^ Several studies have shown that grafted NSCs can survive, proliferate, migrate and exert neuroprotective effects in the brains of PD rat models and that these grafted cells can differentiate into TH‐positive cells in PD rats.^33^, ^34^, ^35^, ^36^, ^37^ However, the issue of the limited migration ability and direction of differentiation of transplanted NSCs needs to be solved.^16^, ^35^ Thus, we focused on promoting the differentiation of NSCs into dopaminergic neurons and the migration of NSCs to the lesion site after transplantation in PD rats.

Based on our previous findings, ethyl stearate can increase TH expression and exert a protective effect on dopaminergic neurons in a PD model.^28^ In this study, we examined that ethyl stearate promoted NSCs differentiation into dopaminergic neurons in vitro experiments. However, it is unclear whether ethyl stearate can promote transplanted NSCs differentiation into dopaminergic neurons in PD rats. The 6‐OHDA‐induced PD model rats exhibited a significant decrease in TH expression and alpha‐synuclein accumulation. Regarding behavioral signs, these model rats rotated more than 6 times per minute after APO injection and exhibited motor deficits including clumsiness in the pole‐climbing test. When NSCs were transplanted combined with ethyl stearate into PD rats, the motor deficits of those rats were alleviated, and the expression of TH was increased. We found that the PD rats that received NSCs transplantation combined with ethyl stearate showed better recovery than those in the NSCs group. In addition, our study showed that ethyl stearate effectively increased TH‐positive cells in both the substantia nigra and the striatum. More grafted cells migrated from the striatum to the substantia nigra and the number of grafted cells was increased in the ethyl stearate group, which illustrated that ethyl stearate may promote transplanted NSCs migration and enhance the survival of grafted NSCs. Thus, the results from this study showed that ethyl stearate can improve the grafted NSCs differentiation into dopaminergic neurons and promote their migration to the lesion, which may be two key steps in ameliorating the behavioral deficits of PD rats.

However, the mechanism by which ethyl stearate promotes the migration of transplanted NSCs is unknown. Chemokines and their receptors were first discovered in the immune system, but it has been frequently demonstrated that chemokines and their receptors are widely expressed in the central nervous system and play an important role in inducing the migration of NSCs.^19^, ^20^, ^21^ Transplanted NSCs exhibit restricted migration capacity, which limits their therapeutic efficacy,^13^ and a low level of expression of some chemokine receptors on NSCs could be an important factor for the slow migration of NSCs.^23^, ^38^ A recent study showed that expressing CCR5 on NSCs accelerated cell migration to the lesion of injury after transplantation in autoimmune encephalomyelitis.^39^ Evidence is emerging that CCL5 and CCR5 are involved in neurological diseases such as multiple sclerosis, stroke, and Alzheimer's disease.^22^, ^40^

Hence, we suspect that ethyl stearate may promote NSCs migration through chemokines and their receptors. The levels of proinflammatory factors and chemokines such as IL‐1β, IL‐33, CCL2, and CCL5 can be increased in PD.^41^ The levels of IL‐15 and CCL5 were increased in the PD patients who received levodopa, compared to healthy controls and the patients with PD.^42^ Our study showed that ethyl stearate apparently improved the migration of NSCs in vitro, and increased the expression of CCR5 on NSCs both in vitro and in vivo. It is worth noting that the expression of CCL5 in the substantia nigra was increased in the ethyl stearate group, which may attract the transplanted cells to migrate to and integrate with the lesion site. We predicted that ethyl stearate might improve the behavioral deficits of PD rats by promoting NSCs differentiation into dopaminergic neurons and the migration of NSCs via CCL5 and CCR5. Indeed, this research requires further experiments. However, ethyl stearate has excellent potential for solving the inadequacy of NSCs transplantation in PD.

## 5 CONCLUSIONS

In summary, CCL5 and CCR5 expression increased significantly after transplantation NSCs combined with ethyl stearate. Ethyl stearate may guide the migration of NSCs by regulating CCL5 and CCR5 and may promote the differentiation of NSCs into dopaminergic neurons in PD model rats.

## AUTHOR CONTRIBUTIONS

Jiapei Huang was responsible for the conceptualization, investigation, validation, and writing of the original draft. Lan Yi reviewed and edited the manuscript. Xiaoxiao Yang investigated the study. Qi Zheng, Jun Zhong, and Sen Ye validated the data. Xican Li contributed resources. Li Hui and Dongfeng Chen supervised the study. Caixia Li conceptualized the study, reviewed, and edited the manuscript, and was involved in project administration. All authors read and approved the final manuscript.

## CONFLICT OF INTEREST STATEMENT

The authors declare no conflicts of interest.

## Data Availability

All data generated or analyzed during this study are included in this published article.
